# Retrospective study of EGFR‐mutant lung adenocarcinoma with bone metastatic clinical features

**DOI:** 10.1002/cnr2.1628

**Published:** 2022-05-25

**Authors:** Liyan Gu, Ting Gong, Qing Ma, Diansheng Zhong

**Affiliations:** ^1^ Department of Medical Oncology Tianjin Medical University General Hospital Tianjin PR China

**Keywords:** bisphosphonate, bone image features, bone metastases, EGFR, lung adenocarcinoma, TKIs

## Abstract

**Background:**

With more and more target medicine application in lung cancer, lots of patients take medicine at home, the treatment bone metastasis and screen of bone metastasis always has been neglected until skeletal‐related events (SREs) such as bone pain, hypercalcemia of malignancy and pathologic fractures emerging which significantly impairs the patients' daily activity ability, seriously lower quality of life.

**Aim:**

To identify the clinical characteristics of patients which influence the overall survival (OS) of EGFR‐TKIs effective in EGFR‐mutant NSCLC with bone metastasis (BM) and the bone metastatic image features.

**Methods:**

We conducted a retrospective study in patients (treated with EGFR‐TKIs ≥6 months) of lung adenocarcinoma with BM in our hospital from October 2014 to October 2017. The Kaplan–Meier survival curves were calculated using the log‐rank univariate test. Multivariate regression analysis was conducted using Cox's regression model. Comparison between the different subgroups of bone metastasis was conducted using Pearson Chi‐Square test.

**Results:**

A total of 79 patients were diagnosed as EGFR‐mutant lung adenocarcinoma with bone metastases. At univariate analysis, age < 65 years (*p* = .024), heavy smoking (*p* = .005), Osteolytic BM (*p* = .034), number of bone metastasis ≥3 (*p* = .032), EGFR‐L858R mutated (*p* = .018) and bisphosphonate times <6 (*p* = .046), were significantly associated with worse overall survival (OS). At multivariate analysis, EGFR 19del was an independent predictor of better OS (*p* = .035). Osteolytic BM was more likely to occur in EGFR‐mutant patients (osteolytic vs. sclerotic vs. mixed: 45.57% vs. 34.18% vs. 20.25%). Patients who had received bisphosphonate ≥6 times were less suffer from SRE compared to those treated with bisphosphonate <6 times (*p* = .019).

**Conclusion:**

In conclusions, this retrospective study suggests that for the patients, treated with EGFR‐TKIs ≥6 months, EGFR exon 19 del, osteogenic bone metastasis, bisphosphonate application times ≥6, smoking <400/day and the number of BM <3 were predictors of better OS (*p* < .05). Bisphosphonate times ≥9 should be considered to the patients with BM. SPECT–CT would be an effective correction of SPECT in the patient's bone metastasis examination. During the whole follow‐up process, we found by chance that the change of bone mineral density in the follow‐up process suggested that bisphosphonates need to be used for more than 1 year or more, and we can use local CT in the follow‐up clinical practice to confirm the bone density changes to decide when we could stop or reduce bisphosphonate application.

## 1 INTRODUCTION

In recent years, the incidence of lung cancer has increased year by year. In 2020, there were 2.2 million new lung cancer patients worldwide, 1.8 million patients died of lung cancer.^1^ Lung adenocarcinoma is currently the most common type of lung cancer. Bone metastases (BMs) is one of the most common metastatic sites, 30%–58% of those with non‐small cell lung cancer develop BMs.^2^, ^3^, ^4^

Bone metastasis is one of the important causes of high mortality in advanced lung cancer and involves skeletal‐related events (SREs) such as bone pain, hypercalcemia of malignancy, pathologic fractures, which significantly impairs the patients' daily activity ability, seriously degrade the quality of life. With more and more targeted drugs clinical applications, such as Epidermal Growth Factor Receptor (EGFR)–Tyrosine Receptor Inhibitors (TKIs), the overall survival time of patients with lung adenocarcinoma, was significantly prolonged.^5^, ^6^, ^7^ The oral taking of targeted drugs allows lung cancer patients to be treated at home, reducing the trouble of commuting to and from the hospital, but also causing some doctors and patients ignoring bone metastasis treatment. The survival prolongation leads to the increase of the incidence of SREs and the aggravation of economic burden. In this context, bisphosphonates, such as zoledronate, the efficient anti‐resorption, are widely used for the treatment and prevention of SREs in lung cancer.^4^, ^8^, ^9^, ^10^, ^11^ They have become the standard treatment for tumor‐induced hypercalcemia and may play an important role in preventing worse of bone metastasis. However, how long for the application of bisphosphonates application is still not clear for patients with BM of lung cancer, especially in patients with BM from lung adenocarcinoma of EGFR‐mutant.

## 2 METHODS

### 2.1 Data collection

A consecutive of EGFR+ (19del and L858R‐mutant) lung adenocarcinoma patients who were histopathologic diagnosis or cytopathologic diagnosis status at Tianjin Medical University General Hospital between October 1st 2014 and October 1st 2017 were included in this study. And the patients had been treated with EGFR‐TKIs for no less than 6 months.

Computed tomography (CT) of chest and upper abdomen, nuclear magnetism (MR) of head and single photon emission computed tomography (SPECT) were performed to each patient to determine tumor‐node‐metastasis (TNM) stages as well as assess the treatment effect according to local procedures every 6–8 weeks. Subsequent CT images were additionally performed on the bone regions where suspicious abnormal skeletal radioactivity uptake had been observed in order to confirm the bone metastasis and the BM character. All the patients' clinical and follow‐up data were analyzed retrospectively.

### 2.2 Diagnosis

SPECT/CT was undergone at nuclear medicine imaging department in Tianjin Medical University General Hospital and diagnosis were made by two radiologists. SPECT scans were over 360° with a duration of 10 s view, imaging matrix, 128 × 128; energy peak, 140 keV; Zoom, 1.0. CT scans were performed with 110 keV and 120 mAs using adaptive dose modulation (CARE Dose 4D). The CT data was reconstructed with 4.8‐mm slice thickness. Syngo software was used to perform fusion images combining SPECT and CT.

Overall survival (OS) was defined as the time from beginning of first‐line treatment to either the date of death or the date of last follow‐up visit. Progression‐free survival (PFS) was defined as the time from beginning of treatment to progression or to death from any cause or the date of last follow‐up visit, whichever occurred first. Patients without tumor progression or death at the time of the data cutoff for the analysis or at the time of receiving additional anticancer therapy were censored at their last date of tumor evaluation.

### 2.3 Statistics

Statistical analysis was conducted by SPSS software (version 22.0). Comparison between the different subgroups of bone‐metastasis patients was conducted using Pearson Chi‐Square test. The overall survival was calculated by Kaplan–Meier method and compared by log‐rank test. Cox survival model was applied to explore the patients' predictors in multivariable analyses. *p* values were two‐sided and *p* < .05 was considered to be statistically significant.

## 3 RESULT

### 3.1 Demographic characteristics

A total of 184 patients were diagnosed as lung adenocarcinoma with advanced stage during the period of study (Table 1). Of these patients, 79 patients (37.5%) got bone metastasis at the time of their initial staging. Female patients had more susceptibility than male to develop the bone metastasis (39.07% vs. 36.87%).

**TABLE 1:** Univariate analysis of survival of lung adenocarcinoma with bone metastasis

		Number	Overall survival (month)		Progression free survival	
			Median (95% CI)	*p* value	Median (95% CI)	*p* value
Gender	Male	43 (54.43%)	23.33 (11.30–29.37)	.166	6.93 (4.34–9.52)	.409
	Female	36 (45.57%)	25.90 (18.17–35.64)		11.97 (6.96–16.97)	
Age	≥65	40 (50.63%)	23.90 (16.59–27.21)	.124	11.27 (6.25–16.28)	.005
	<65	39 (49.37%)	29.27 (15.44–36.10)		6.90 (4.45–9.35)	
Smoke index	≥0, <400	54 (68.35%)	26.90 (16.90–35.90)	.005	14.23 (7.89–20.57)	.001
	≥400	25 (31.65%)	19.03 (14.07–24.00)		9.03 (6.18–11.88)	
EGFR mutant	19del	45 (56.96%)	31.17 (11.70–43.36)	.018	16.53 (13.22–19.85)	.058
	L858R	34 (43.04%)	22.46 (10.81–32.99)		9.87 (0.00–28.88)	
Number of BM	<3	36 (45.57%)	36.62 (28.84–44.40)	.002	13.57 (10.98–16.16)	.070
	≥3	43 (54.43%)	20.90 (16.79–26.50)		5.70 (1.96–9.44)	
SRE	Yes	57 (72.15%)	21.87 (14.44–25.29)	.103	7.87 (5.01–10.73)	.866
	No	22 (27.85%)	25.27 (18.13–38.40)		10.37 (8.22–12.52)	
Bisphosphonate times	<6	41 (51.90%)	19.40 (14.16–24.33)	.038	6.00 (2.03–9.97)	.036
	≥6	38 (48.10%)	31.16 (24.63–38.17)		11.13 (4.15–18.12)	
BM characteristics	Sclerotic	27 (34.18%)	32.19 (19.45–39.76)	.034	14.53 (8.69–20.38)	.099
	Osteolytic	36 (45.57%)	16.80 (14.87–24.73)		6.93 (2.26–11.61)	
	Mix	16 (20.25%)	20.03 (14.69–28.11)		7.87 (5.91–9.82)	

Among the 79 lung adenocarcinoma patients with bone metastasis, 35 (44.30%) patients suffered at least one SRE. Six hundred twenty‐three SPECT examinations related to bone were performed on those 79 patients, 1564 CTs, 11 MRs, 15 PET‐CTs respectively. Among all these images, confirmation scans including SPECT–CTs and MRs were performed on those whose SPECT results showed suspicious abnormal skeletal radioactivity uptake. Thirteen of these confirmative images (11 SPECT–CTs and 2 MRs) showed partially or completely inconsistent with SPECT, the error rate of SPECT was 5.42%.

Some cases of bone metastasis involved multiple locations, and a total of 182 metastatic locations were recorded at the first time diagnosis of the bone metastasis: Spine was the most common site (*n* = 83, 45.60%), and 28 (15.38%) in thoracic, 23 (12.64%) in sacrum, 21 (12.57%) in lumber, 11 (6.04%) in cervical, respectively. The other sites of bone were the place most frequent metastasis to were ribs (*n* = 27, 14.84%), pelvis (*n* = 23, 12.64%), scapula (*n* = 17, 9.34%), femur (*n* = 14, 7.70%), humerus (*n* = 10, 5.49%) and skull (*n* = 8, 4.40%).

The clinical parameters and OS analysis of patients are displayed in Table 1.

**FIGURE 1:**
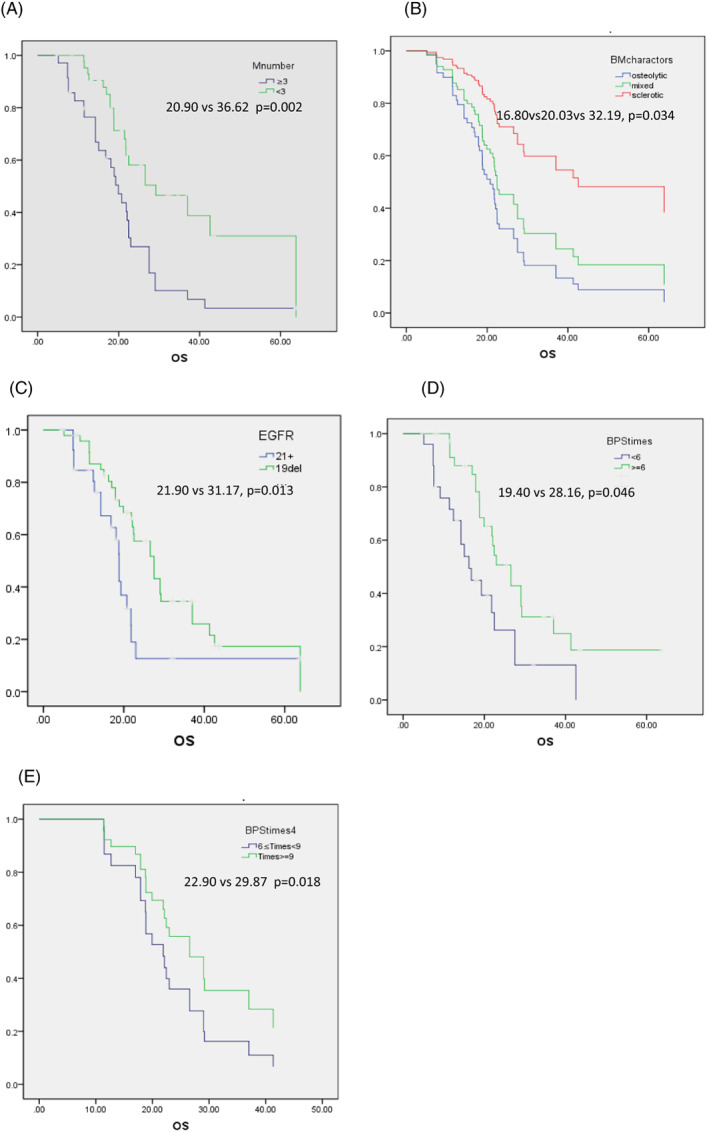
Kaplan–Meier plots of OS (month) of lung adenocarcinoma patients with BM according to (A) BM numbers, (B) characters of BM, (C) EGFR mutation sites, (D) times of bisphosphonate (times <6 or times ≥6), (E) times of bisphosphonate (≥6 and <9 or times ≥9). Median OS are expressed in months. Statistical significance was assessed by the log‐rank test.

The OS of patients aged <65 was shorter than the patients aged ≥65 (19.27 vs. 23.9 months, *p* = .124). Based on the smoking index, heavy smokers (≥400) had worse OS (19.03 vs. 26.90 months, *p* = .005) compared with mild smokers. Stratified by number of BMs, median OS was 36.62 months (95% CI 28.84–44.40 months) in BM number <3 and 20.90 months (95% CI 16.79–26.50 months) in BM number ≥3 (*p* = .002) (Figure 1A). Among the three different BM characteristics, it was found that Median OS was 16.80 months (95% CI 14.87 to 24.73) in osteolytic, 29.19 months (95% CI 19.45 to 39.76) in sclerotic type and 20.03 months (95% CI 14.69 to 28.11) in mixed type (*p* = .034) (Figure 1B).

For the EGFR mutant lung adenocarcinoma with BM, the OS of EGFR 19del patients are is longer than of EGFR L858R mutant ones (31.17 vs. 21.90 mo, *p* = .013) (Figure 1C). However, the difference of PFS between the two groups showed no significance (16.53 vs. 9.87 mo, *p* = .358). At univariate analysis, age < 65 years, heavy smokers, EGFR L858R mutant, number of bone metastasis ≥3, bisphosphonate times <6, and osteogenic BM were significantly associated with worse OS. At multivariate analysis, only EGFR 19del was an independent predictor of OS (*p* = .035).

We found that the EGFR mutant site had some relation with the BM characteristics. The results showed that osteolytic type of BM was more likely to occur in EGFR19del patients (*p* = .004). But there was no significant difference between BM number and EGFR mutant site. Besides, when it comes to the bisphosphonate application, it showed that patients who were treated with bisphosphonate ≥6 times were less likely to suffer from SRE compared to the ones bisphosphonate application <6 times (*p* = .019).

### 3.2 Safety

The average time was 7.9 months (3–22 months) of all the patients applied bisphosphonates, only one case of vomiting was Grade 3, the others were Grade 1–2. Among them, 10 cases of fever (≥38°C) lasted 1–7 days, 4 cases of vomiting, 3 cases of bone pain, and 5 cases of fatigue. There was no side effect of mandibular necrosis.

## 4 DISCUSSION

It took about 10 years that the median over survival (mOS) of IIIB‐IV stage non‐small cell lung cancer increased from 8 ~ 12 months to 15 ~ 17 months.^6^, ^7^, ^12^ In addition, with the EGFR‐TKIs application, the mOS was prolonged to more than 30 months for EGFR sensitizing mutated NSCLC.^13^ The risk of bone metastasis in advanced lung adenocarcinoma is increased. Some research from Asia^2^, ^14^ showed the incidence of bone metastasis in advanced stage lung cancer was about 30%. Besides, in the largest of the earlier studies, using the linked Surveillance, Epidemiology, and End Results (SEER)‐Medicare database in the United States, Sathiakumar et al.^15^ found that 38% of 59 319 distant metastasis lung cancer patients aged ≥65 years had claims‐based evidence of bone metastasis. The downsides along with SPECT examination were the inaccuracy and less specificity. SPECT–CT was approved as a useful complement over SPECT, which decreased the equivocal lesions to 8%–22%.^16^, ^17^ In our study, CT tomography was performed on uncertain BM lesions to confirm BM, so the lower error rate 5.42% was based on the objective judged reconfirm SPECT results.

During early steps of bone metastatic colonization, tumor cells infiltrate the osseous fenestrated capillary bed, which offers little mechanical constraint to tumor cells that engage with the host microenvironment.^18^ In this study, spine was the most common site of bone metastasis, followed by ribs and pelvis. This finding was in consistent with previous reports.^19^, ^20^ Possible mechanism was the access of abundant blood supply from plexus vertebral system in vertebral bodies in the thoracic and lumbar spine and the high bone marrow flow of some skeletal elements.

When lung cancer cells reach local bone tissue, lung cancer cells secrete a variety of cytokines to stimulate the production of pathological osteoclasts, and activate osteoclasts to increase osteolytic bone resorption and bone destruction, which leads to skeletal‐related events (skeletal‐related events, SREs), including bone pain, hypercalcemia, etc. and a significant decline in the life quality of the patients. One of the purposes of treating lung adenocarcinoma patients with bone metastasis is to improve life quality of patients, relieve pain symptoms and prevent or reduce complications, such as pathological fracture and spinal cord compression symptoms. A retrospective study^21^, including 29 720 lung cancer patients, showed that the patients with both bone metastases and at least one SRE, the >6‐month mortality rate was nearly three times as high as that among lung cancer patients with no bone metastases. The >2‐month mortality rate was approximately twice as high as in patients with both bone metastases and at least one SRE compared with lung cancer patients with bone metastases but no SREs. Our results did not indicate the relevance between SREs and overall survival in lung adenocarcinoma patients. Part from the reason of selection bias, however, in this study an important pitfall was the fact that the initiation of bisphosphonate was performed the time of diagnosis, which means the treatment for the bone metastasis precede SREs in most cases, so that causal relationships between SREs and OS were probably disguised. As it also showed in our present results, patients whose bisphosphonate application ≥6 times, have a longer OS (19.40 m vs. 28.16 m, *p* = .039) (Figure 1D) and a lower risk of SREs (*p* = .019) compared to patients bisphosphonate application <6 times. This proved our inference laterally. We further analyzed that bisphosphonates were applied more than 9 times, the OS had been further prolonged than the patient who had accepted ≥6 and <9 times (29.87 m vs. 22.9 m, *p* = .018) (Figure 1E). Therefore, we believe that the continuous application of bisphosphonates in TKIs long‐term treatment patients of EGFR mutant lung cancer with BM is necessary in prolonging survival time. And no adverse effects of mandibular necrosis were observed.

In contrast to the lower occurrence in Caucasian population (10%), mutations in the tyrosine kinase domain of the EGFR gene are most often found in Asians (30–50%),^22^ which constitute independent favorable predictive factors in EGFR TKIs therapy.^23^, ^24^ Fewer data were found with regard to the population of lung cancer patients with BM. In this study, we found that the patients with EGFR 19 del, mOS was longer than the patients with EGFR L858R mutant (*p* = .047), which was also similar to previous data of overall population from lung adenocarcinoma.^25^

Of three types of bone metastasis: osteolytic, osteoblastic and mixed lesion, the osteolytic is dominant in patients with non‐small cell lung cancer, while small cell lung carcinoma most likely to contain osteoblastic lesions.^26^ In our study, the percentage of osteolytic is 45.37% and mixed lesion is 20.45%, which are more different than previous reports. We think the main reason is that when we assess the bone metastasis through ECT each focus would be given CT tomography again. So the lesion character was much more accurate. We also found that the OS of osteoblastic BM is longer than the other two types (*p =* .034). An interesting point of our investigation is that differentiation of BM feature was harbored in different EGFR status groups (*p* = .004), which was little elucidated in the former publications. A previous retrospective study^27^ suggested a correlation between the CT imaging‐based histogram features of bone metastases and their EGFR mutation status; it also suggested that the CT histogram features were the biomarker of EGFR. BMs with EGFR mutation tended to have a higher level of intratumoral non‐uniformity compared with bone metastases without EGFR mutation. Whether this difference is caused by the type of bone metastasis, it may need to be further confirmed by larger samples.

During the whole follow‐up process, we found by chance that the increase of bone mineral density in radiological images of patients with stable lytic bone metastasis occurred after 6 months or later after regular application of bisphosphonates, with the latest case appearing at 14 months, and the increase of bone density did not reach 50% of the normal bone density till 1 year bisphosphonates application. However, no further increase in bone density was observed after the application of bisphosphonates in osteoblastic bone metastasis. The possible mechanism of BPs is that the inorganic components bound to bone and act on osteoclasts, and BPs will be released the place of bone absorption, which can induce apoptosis in osteoclasts.^28^ BPs can also act on osteoblasts, inhibit their secretion of osteoclast stimulating factor, or promote their secretion of osteoblast inhibiting factor, thus indirectly inhibiting bone absorption. With osteoclast apoptosis, bone resorption and bone remodeling decrease, which increases and maintains bone mass and reduces the risk of fracture.^29^

Therefore, it is necessary to use bisphosphonates for a long time no less than 1 year. BPs can reduce the risk of fractures by reducing bone turnover to slow bone loss and increase bone density. However, as the above observations were found in the chest CT of follow‐up, more rigorous bone CT should be used in the later work to observe the changes of bone trabeculae and the bone matrix, so as to clarify the whole change process and play a further guiding role for the application of bisphosphonates.

## 5 CONCLUSION

In conclusion, this retrospective study had indicated that exon‐19del, osteogenic bone metastasis and bisphosphonate application times ≥6 were predictors of better OS for the TKIs effective patients. Bisphosphonate application ≥9 times should be considered for the patients with bone metastasis. SPECT–CT would be an effective correction of SPECT in the patient's bone metastasis examination. During the whole follow‐up process, we found by chance that the change of bone mineral density in the follow‐up process suggested that bisphosphonates need to be used for more than 1 year or more, and local CT could be used in the follow‐up clinical practice to confirm the bone mineral density and then we could stop or reduce bisphosphonate application.

## AUTHOR CONTRIBUTIONS

**Liyan Gu:** Conceptualization (lead); data curation (lead); formal analysis (lead); methodology (lead); project administration (lead); resources (lead); software (lead); supervision (supporting); validation (lead); writing – original draft (lead). **Ting Gong:** Data curation (supporting); formal analysis (supporting); investigation (supporting); project administration (supporting); resources (supporting). **Qing Ma:** Data curation (supporting); formal analysis (supporting). **Diansheng Zhong:** Conceptualization (supporting); data curation (supporting); formal analysis (equal); funding acquisition (lead); methodology (equal); project administration (supporting); supervision (lead); validation (lead); writing – original draft (supporting); writing – review and editing (lead).

## CONFLICT OF INTEREST

The authors have stated explicitly that there are no conflicts of interest in connection with this article.

## ETHICS STATEMENT

Ethics committee approval was obtained for publication of this retrospective data: Tianjin Medical University General Hospital Ethics Review Committee. The requirement for consent was waived.

## Data Availability

The processed data required to reproduce these findings cannot be shared at this time as the data also forms part of an ongoing study.
